# Mouth Breathing and Craniofacial Development in Children: A Systematic Narrative Review and Clinical Implications

**DOI:** 10.3390/healthcare14121737

**Published:** 2026-06-16

**Authors:** Elizabeth Sotero Grande, Ximena Alejandra Checa-Caratachea, Luis Pablo Cruz-Hervert, Gustavo Castillo Salazar, Álvaro Edgar González-Aragón Pineda

**Affiliations:** 1 Bioprogressive Institute of Orthodontics, Avenida de Los Deportes Number 14, Las Arboledas, Tlalnepantla de Baz 54026, Mexico; elysg04@gmail.com (E.S.G.);; 2 Faculty of Higher Studies Iztacala, National Autonomous University of Mexico, Avenida de los Barrios Number 1, Los Reyes Ixtacala, Tlalnepantla de Baz 54090, Mexico; 3 Graduate and Research Division at the School of Dentistry, National Autonomous University of Mexico, Ciudad de México 04510, Mexico

**Keywords:** mouth breathing, craniofacial growth, maxillofacial development, malocclusion, airway obstruction, children

## Abstract

**Highlights:**

**What are the main findings?**

- Mouth breathing is consistently associated with craniofacial alterations in children, including maxillary narrowing, increased vertical growth patterns, and a higher prevalence of malocclusions.
- Cephalometric findings suggest structural differences between mouth and nasal breathers; however, evidence is heterogeneous and largely based on observational studies.

**What are the implications of the main findings?**

- Mouth breathing should be considered a relevant functional factor in craniofacial development, requiring early detection and interdisciplinary management.
- Current evidence does not support a definitive causal relationship, highlighting the need for standardized and longitudinal studies.

**Abstract:**

**Background/Objectives**: Mouth breathing in childhood has been associated with alterations in craniofacial growth and the development of the maxillofacial complex. However, arguments persist regarding the scale of this relationship and its clinical consequences because of the diverse nature of the available evidence. This review aims to evaluate the evidence on the relationship between mouth breathing and maxillofacial development in children. **Methods**: A systematic narrative review with qualitative synthesis was conducted using databases such as PubMed/MEDLINE, SciELO, Cochrane Library, Google Scholar, and institutional academic repositories. Studies in English and Spanish that evaluated mouth breathing and its impact on craniofacial growth in children were included. The selection process involved reviewing titles, abstracts, and full texts against set inclusion and exclusion criteria. **Results**: The analyzed studies show a consistent association between mouth breathing and maxillofacial developmental abnormalities, including maxillary narrowing, high-arched palate, increased vertical growth pattern, and a higher prevalence of malocclusions. Likewise, considerable heterogeneity was observed in the diagnostic criteria used to define mouth breathing and to evaluate craniofacial development among the included studies. Furthermore, characteristic cephalometric changes were identified in patients with mouth breathing compared to nasal breathers. **Conclusions**: Evidence suggests a consistent association between mouth breathing and craniofacial alterations in children, including structural and dentofacial changes. Nonetheless, the variety in methods and the reliance on observational data restrict the ability to confirm definitive cause-and-effect links. Early identification and interdisciplinary management may help reduce the progression and severity of associated craniofacial alterations.

## 1. Introduction

Breathing is an essential physiological function for maintaining homeostasis and the proper development of the organism. Under normal conditions, nasal breathing allows filtration, humidification, and thermal regulation of inhaled air. Furthermore, it promotes functional harmony among craniofacial structures, supporting proper craniofacial growth through mastication and swallowing [1,2,3,4]. However, when this pattern is altered and replaced by mouth breathing, it can be associated with alterations in the growth of the maxillofacial complex [5,6].

Nasal breathing also promotes a proper functional balance of the orofacial tissues and the craniofacial complex. However, mouth breathing can become a compensatory mechanism for inadequate ventilation when the upper airway is partially or completely blocked [1,2]. Clinically, chronic mouth breathing has been associated with facial features known as “adenoid facies,” including lip incompetence, increased lower facial volume, a narrow palate, and a vertical growth pattern. Furthermore, some authors have noted a possible relationship between mouth breathing, sleep-disordered breathing, and pediatric obstructive sleep apnea, conditions that can affect children’s growth and development [5,7].

Mouth breathing in childhood has been widely associated with changes in craniofacial development, including transverse narrowing of the maxilla, a high or pointed palate, increased vertical growth pattern, and a higher prevalence of malocclusions [5,6,8,9]. Beyond aesthetics, these alterations affect core functions such as mastication, deglutition, phonation, and speech, leading to changes in posture and dental alignment, thereby diminishing the quality of life for pediatric patients [9,10,11].

From a pathophysiological perspective, several mechanisms have been proposed to explain this association. Mouth breathing is often accompanied by changes in craniocervical posture, tongue thrust, lip incompetence, and alterations in the balance of perioral muscle forces. These functional changes reduce lateral stimulation of the maxilla, favoring its transverse collapse and the development of dentofacial alterations [4,8,9,12]. Additionally, conditions such as adenoid hypertrophy, allergic rhinitis, and other forms of upper airway obstruction act as perpetuating factors related to mouth breathing [1,2,3,9,10].

Despite the clinical consistency of these observations, scientific literature shows heterogeneity in study designs, diagnostic criteria, and variables analyzed, which limits the interpretation of the strength of the association and the ability to establish causal relationships. Some studies have reported significant cephalometric differences between mouth and nasal breathers, while others point to the influence of confounding factors such as genetics, oral habits, and the patient’s functional environment [5,6,8,9].

In this context, it is necessary to integrate the available evidence through a systematic narrative review to synthesize existing findings, identify consistent patterns, and recognize current methodological limitations. It is also essential to highlight the clinical implications of this relationship, particularly in terms of early diagnosis and an interdisciplinary approach. Therefore, this study aims to examine scientific findings on how mouth breathing in children affects their craniofacial development and dentofacial alterations.

## 2. Materials and Methods

### 2.1. Study Design

A systematic narrative review was conducted to analyze the available evidence on the relationship between mouth breathing and maxillofacial development in children. The literature search was conducted between August 2025 and March 2026 in five electronic databases: PubMed/MEDLINE, SciELO, the Cochrane Library, the institutional repository of the National Autonomous University of Mexico (UNAM), and Google Scholar. Medical Subject Headings (MeSH) and keywords were used to identify studies, including “Mouth Breathing”, “Oral Breathing”, “Maxillary Development”, “Craniofacial Growth”, “Maxillofacial Complex”, and “Malocclusion”. The terms were combined using Boolean operators (AND, OR) to optimize the retrieval of relevant studies. Additionally, a manual search of the reference lists of the selected articles was conducted to identify further studies of interest.

The search strategy was adapted to the indexing terms of each database. The complete and reproducible search strings used were:

- (“Mouth Breathing” OR “oral breathing”);
- AND (“Maxillary Development” OR “Craniofacial Growth” OR “Maxillofacial Complex”);
- AND (“Malocclusion” OR “Dentofacial abnormalities”).

Table 1 outlines the key features of the search approach, selection standards, and methodological process for this review.

Specific filters were applied to the database, including publication dates from January 2000 to March 2026, in English and Spanish, and with full-text availability.

### 2.2. Selection Criteria

Original studies published in English or Spanish were included, regardless of publication date. These studies covered observational, cross-sectional, case–control, cohort, case report, systematic review, and meta-analysis designs. The research specifically addressed mouth breathing and its consequences for the maxillofacial development of children.

A broad range of study designs was included to provide a comprehensive overview of the available evidence, including systematic reviews, meta-analyses, and clinical studies, in order to synthesize current knowledge on the topic. Studies presented as conference papers, letters to the editor, posters, or incomplete findings were excluded.

### 2.3. Study Selection Process

The search, study selection, and data extraction processes were primarily conducted by one reviewer. The eligibility of the included studies was independently verified by a second reviewer, and any discrepancies were resolved by consensus. The initial search yielded a considerable number of potentially relevant publications, but multiple studies were subsequently excluded due to reasons such as duplication, incomplete data, the absence of specific craniofacial assessment, or failure to adhere to the methodological criteria set for this review. This systematic narrative review was conducted following the general principles of the 2020 PRISMA statement, adapted to the qualitative nature and heterogeneity of the included studies. Duplicate records were identified manually by comparing titles, authors, and year of publication before conducting the final study selection process.

Due to the heterogeneity of the included studies in terms of design, populations, and outcome measures, a qualitative synthesis was performed. A formal risk of bias assessment was not conducted, which is acknowledged as a limitation of this review.

## 3. Results

**Figure 1:**
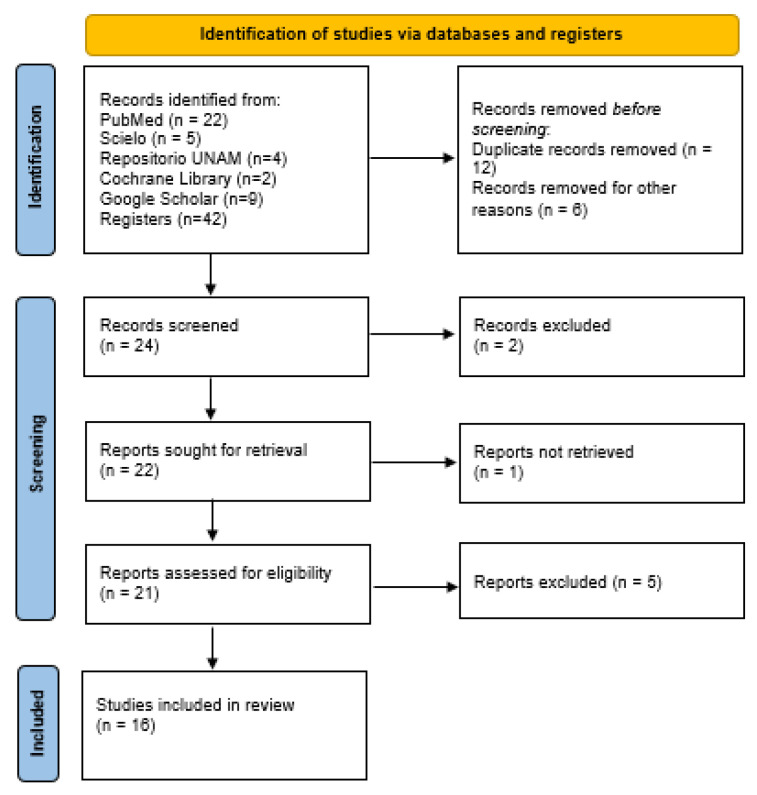
PRISMA flow diagram of the study selection process.

Initially, 42 potentially relevant records were found in the databases searched. After removing 12 duplicate records and 6 records excluded because they showed incomplete results, 24 studies underwent the screening process. Subsequently, 2 records were excluded during this stage, 1 report could not be retrieved for full evaluation, and 5 additional studies were excluded after the eligibility assessment. Finally, 16 studies were included in this systematic narrative review. The study selection process is presented in Figure 1.

### 3.1. Characteristics of the Included Studies

The selected studies exhibit heterogeneity in methodological design, including systematic reviews with meta-analysis, observational studies, cross-sectional studies, and clinical reports, with observational studies and systematic reviews predominating. Some included studies were systematic reviews that synthesized multiple primary studies. Most studies evaluated children and used cephalometric, clinical, and functional variables to analyze the relationship between mouth breathing and craniofacial development. It is important to acknowledge that certain systematic reviews combine several primary studies, enabling a wider understanding of the evidence than what individual studies alone offer.

Regarding methodological approaches, some studies performed quantitative analyses using meta-analysis, while others employed clinical evaluations, observational records, and descriptive analyses. This methodological diversity reflects the complexity of the phenomenon studied. The main characteristics of the included studies are summarized in Table 2. Due to the methodological heterogeneity of the included studies, the reported variables and diagnostic criteria used to define mouth breathing varied considerably between investigations. Therefore, the findings were summarized using descriptive statistics, focusing on comparing the main craniofacial alterations and cephalometric data observed.

### 3.2. Craniofacial Alterations Associated with Mouth Breathing

According to the studies, mouth breathing correlates with alterations in the development of the maxillofacial complex. Among the main characteristics reported are transverse narrowing of the maxilla, a high or ogival palate, an anterior open bite, a posterior crossbite, and an increased vertical facial growth pattern [14,18,19]. These alterations were described in both clinical studies and cephalometric analyses, revealing a characteristic dentofacial pattern in patients with mouth breathing.

### 3.3. Cephalometric Findings

Significant disparities were observed in studies utilizing cephalometric analysis when comparing mouth and nasal breathers. A decrease in SNA and SNB values, an increase in the ANB angle, an increase in parameters related to vertical growth (SN-PP, SNGoGn), and a reduction in airway dimensions were observed [5]. The findings indicate that mouth breathers tend to have maxillary and mandibular retrusion and exhibit an increased vertical growth pattern.

### 3.4. Factors Associated with Mouth Breathing

Several studies have identified etiological and associated factors for mouth breathing, including adenoid and tonsillar hypertrophy, allergic rhinitis, and chronic upper airway obstruction [1,9,10]. These factors contribute to the establishment and maintenance of the mouth-breathing pattern, favoring the development of functional and structural alterations.

### 3.5. Summary of the Evidence

Most of the studies examined indicate a steady connection between mouth breathing and alterations in craniofacial development. Still, variations in diagnostic standards, analyzed elements, and research approaches impede straightforward comparisons across studies. Even with these drawbacks, the current information indicates that the breathing pattern is a significant functional element in maxillofacial development.

## 4. Discussion

This systematic narrative review demonstrates a consistent association between mouth breathing and alterations in the development of the maxillofacial complex in children. According to the studies, this breathing pattern correlates with structural and functional alterations, notably maxillary narrowing, increased vertical growth, and malocclusions.

One of the most relevant findings corresponds to the cephalometric alterations described in the literature. Increased vertical growth, a narrowed maxilla, and facial characteristics resembling adenoid facies with functional respiratory issues are among the most commonly noted craniofacial and cephalometric alterations [6,16]. The meta-analysis by Zhao et al. reports that patients with mouth breathing showed lower SNA and SNB values, along with an increased ANB angle, suggesting a tendency toward maxillary and mandibular retrusion. Likewise, an increase in parameters related to vertical growth was observed, which is consistent with the dolichofacial pattern frequently described in these patients [5]. These results are consistent with observational studies such as those by García et al., Paolantonio et al., and Valdés et al., who reported a high prevalence of narrow palates and anterior open bites in children with respiratory disorders [6,16,19], findings that are consistent with more recent studies [24,25]. These findings support the evidence synthesized in the included systematic reviews, as these reviews combine several investigations yielding comparable outcomes.

Noor et al. reported a higher percentage of malocclusion in mouth-breathing subjects [20], which agrees with the findings of Festa et al. and Morales et al., who suggest that malocclusion may play an important role in the development of mouth breathing or be a consequence of it [15,21]. However, most of these findings are derived from observational studies, which limits the ability to establish causal relationships. In contrast, these results differ from those reported by Souki et al., who found no significant association between malocclusion and mouth breathing [14], and from those observed by Morales et al., who reported a higher frequency of malocclusion in nasal breathers compared to mouth breathers [15]. In this regard, recent documents, such as the white paper by the American Association of Orthodontists [7,26], have indicated that the relationship between craniofacial characteristics and sleep-disordered breathing is complex and not necessarily causal, with inconsistent or inconclusive results reported in the literature. This reinforces the need for caution in interpreting the association between mouth breathing and craniofacial development, considering the multifactorial nature of these processes. The discrepancies may be due to differing population ages, airway obstruction severity, individual growth, oral habits, and the criteria used to define mouth breathing.

From a pathophysiological perspective, these findings may be explained by alterations in orofacial muscle balance. Mouth breathing correlates with a low, forward tongue posture and reduced lip muscle tone, which diminishes the lateral forces vital for maxillary transverse development. Therefore, the breathing pattern might not just be a result of blocked airways but could also be linked to alterations in structural development [12].

On the other hand, some of the analyzed studies provide evidence on therapeutic interventions and their impact on respiratory function. The systematic review on rapid maxillary expansion suggests that this treatment can improve breathing in patients with mouth-breathing patterns by increasing the volume of the nasal cavity and facilitating airflow [22]. However, the long-term benefits are inconclusive, highlighting the need for longitudinal studies to evaluate the stability of these changes. Similarly, the review on microimplant-assisted maxillary expansion (MARPE) reports significant structural changes in the craniofacial complex, although it emphasizes the need for further evidence to confirm these effects [8].

Despite some studies reporting improvements in airway dimensions and breathing patterns following maxillary expansion procedures, there is an ongoing debate about the long-term stability and clinical relevance of these changes. Therefore, these interventions should not be considered definitive solutions to the underlying functional problem but rather as part of a multidisciplinary approach [7,22].

Also, despite the consistency of the findings, the available evidence has significant limitations. There is considerable heterogeneity in the diagnostic criteria for mouth breathing, as well as in the methodologies used to assess craniofacial growth. Some studies relied on clinical evaluations for diagnosing mouth breathing; others incorporated otolaryngological findings, cephalometric analyses, questionnaires, or functional assessments. The absence of consistent diagnostic criteria could be a contributing factor to the varying and conflicting findings across the studies. Furthermore, most of the studies are observational designs or reviews, which limit the ability to establish causal relationships. The presence of confounding factors, such as genetics, oral habits, and environmental conditions, also complicates the interpretation of the results. Additionally, while observational studies and case reports offer valuable clinical insights, their evidence level is lower, potentially affecting the study’s methodological strength and the applicability of its results. Furthermore, a formal assessment of potential bias was not conducted with standardized instruments because of the considerable variation in study methodologies, participant groups, and analyzed variables across the included research. This situation could lead to less methodological rigor in the review, less robust conclusions, and a need for cautious interpretation of the findings.

### 4.1. Strengths and Limitations of the Review

Key strengths of this review include its inclusion of up-to-date evidence and a qualitative synthesis of clinical, cephalometric, and functional aspects of mouth breathing in children. In addition, it features diverse diagnostic and therapeutic strategies, enabling a wider understanding of the craniofacial alterations connected to this disorder.

A key contribution of this review is its integrative approach. A comprehensive perspective on the potential influence of mouth breathing on infant craniofacial development is presented, drawing on clinical, functional, cephalometric, and airway-related evidence. It also integrates studies with diverse methodological designs and highlights the need for more standardized diagnostic criteria to strengthen the available evidence and guide future research.

The lack of analytical studies or meta-analyses should be considered during the interpretation of the results. Moreover, longitudinal studies are rare, making it difficult to assess how breathing patterns change and affect craniofacial growth throughout development. Additionally, some included studies have small sample sizes or lack standardization in cephalometric measurements, which may affect the external validity of the results.

Another limitation of this review is the relatively small number of included studies. This may be related to the strict eligibility criteria applied, the specific focus on pediatric populations, and the variability in diagnostic definitions of mouth breathing and in the variables used to assess craniofacial development. Therefore, the data are scarce, and the results need to be viewed with reservation.

### 4.2. Clinical Implications

From a clinical perspective, the findings of this review highlight the importance of early diagnosis of mouth breathing in children, as some cases may be misinterpreted or associated with conditions such as attention deficit hyperactivity disorder (ADHD) related to sleep disturbances secondary to respiratory obstructions [27]. Chronic mouth breathing has been linked in several studies to sleep-disordered breathing, including obstructive sleep apnea in children, which can negatively influence their growth, academic success, and quality of life [7].

Likewise, certain authors have observed an increased occurrence of dental cavities and disruptions in saliva equilibrium among mouth-breathers. This is likely due to decreased saliva production and alterations within the oral cavity [28]. Timely identification of this breathing pattern allows for the implementation of interdisciplinary interventions that include orthodontics, otolaryngology, and myofunctional therapy, with the aim of preventing more severe structural alterations and improving the stability of treatments.

Overall, the evidence analyzed suggests that mouth breathing is consistently associated with craniofacial and dentofacial alterations in the pediatric population, including vertical growth patterns, maxillary narrowing, and malocclusions. However, studies with more robust methodological designs and standardized criteria are needed to clarify the nature of this association and strengthen the available evidence.

## 5. Conclusions

Mouth breathing appears to be consistently associated with craniofacial and various dentofacial abnormalities in the pediatric population. The available evidence indicates that mouth breathing is connected to modifications in the development of the maxilla, the placement of the mandible, and occlusal patterns. However, due to the heterogeneity of the included studies and the predominance of observational evidence, a definitive causal relationship cannot be established.

Early diagnosis and interdisciplinary management of mouth breathing may help prevent or reduce the severity of associated craniofacial alterations.

## Figures and Tables

**Table 1:** Summary of the search strategy and selection criteria.

Parameter	Description
Databases consulted	PubMed/MEDLINE, SciELO, Cochrane Library, Google Scholar and institutional repositories
Search timeframe	August 2025 to March 2026
Languages included	English and Spanish
Keywords	“mouth breathing”, “oral breathing”, “craniofacial development”, “malocclusion”, “maxillary constriction”
Boolean operators	AND/OR
Types of studies included	Observational studies, systematic reviews, meta-analyses, cross-sectional studies, and case reports
Target population	Pediatric patients with mouth breathing
Variables analyzed	Craniofacial alterations, vertical growth, malocclusions, cephalometric findings
Exclusion criteria	Letters to the editor, conference abstracts, incomplete studies or studies without relevant results
Selection process	Primary review by one evaluator and independent verification by a second reviewer

**Table 2:** Characteristics of the included studies.

Reference	Type of Study	Age of the Patients	Main Variable	Main Findings
Mattar et al., 2004 [13]	Observational.	Children aged 3 to 6 years.	Skeletal and occlusal characteristics (tonsillar hypertrophy, rhinoscopy, lateral skull radiograph and cephalometric tracings evaluating the angles SNA, SNB, ANB, SN.GoGn, SN.PP, PP.MP. ArGo. GoMe, SNGn, BaN.PtGn and the linear measurements N-Me, N-ANS, ANS-Me. S-Go, S-Ar and Ar-Go) and mouth breathing.	A significant difference was found in the values of SN.GoGn, PP.MP BaN.PtGn, Ar-Go, and S-Go in mouth-breathing children. The intermolar distance was smaller in mouth-breathers.
García et al., 2007 [6]	Descriptive observational.	Children aged 6 to 12 years.	Relationship between Nasal Respiratory Insufficiency and Dental Malocclusions.	Despite no statistically significant association between nasal respiratory insufficiency and specific malocclusion types, dentofacial characteristics linked to mouth breathing were identified, primarily Class I malocclusion (61%), narrow palates (67%), and anterior open bites (50%) in children.
Souki et al., 2009 [14].	Cross-sectional.	Children aged 2 to 12 years.	Malocclusions and mouth breathing.	No significant association was found between the severity of airway obstruction (adenoid/tonsillar hypertrophy or rhinitis) and Class II malocclusion, anterior open bite, or posterior crossbite.
Morales et al., 2009 [15]	Cross-sectional.	Children aged 9 to 16 years.	Malocclusions and mouth breathing.	Among mouth breathers, skeletal Class II was the most frequent pattern, whereas Class I and Class III malocclusions were more commonly observed among nasal breathers.
Valdés et al., 2013 [16].	Cross-sectional.	Children aged 9 to 12 years.	Malocclusions, palatal depth and facial type associated with mouth breathing.	In mouth breathers, a prevalence of malocclusions of 98.1% was found, with Class II predominating at 55.6%. In addition, the presence of an anterior open bite was found in 9.3% and a posterior crossbite in 7.4%. Significant differences were found in the measurement of overjet, palatal depth, and malocclusions in mouth breathers.
Lione et al., 2014 [17].	Cross-sectional.	Children.	Dimensions of the maxillary arch (anterior arch length (AAL), total arch length (TAL), palatal morphology using dental models and mouth breathing.	No difference was found in the sagittal AAL and TAL measurements. The height of the palatal vault was greater in mouth breathers at the level of the second deciduous molars and first permanent molars, and smaller angular measurements were found in mouth breathers.
Borborema et al., 2015 [18].	Case report.	A 16-year-old teenager.	Partial glossectomy in a patient with class III malocclusion, maxillary hypoplasia, dental crowding, open bite and respiratory difficulty associated with macroglossia.	Macroglossia compromised the airway and was associated with muscle hypertrophy, mandibular prognathism, and growth of the maxillofacial complex, while glossectomy decreased airway compromise.
Paolantonio et al., 2019 [19]	Cross-sectional.	Children aged 3 to 6 years.	Malocclusions and mouth breathing.	The prevalence of mouth breathing increases with the severity of malocclusion, and mouth breathing is closely related to anterior open bite, posterior crossbite, and incisor overjet.
Zhao et al., 2021 [5].	Systematic review with meta-analysis.	Children <18 years old with maxillofacial deformities associated with mouth breathing.	Effect of mouth breathing on skeletal development and malocclusion.	Mouth-breathing children exhibited reduced SNA and SNB values. ANB, 1,NA, 1-NA, 1-NB, and the SN-PP, SN-OP, PP-MP, and SNGoGN indices were higher in mouth breathers compared to nasal breathers. Airway data from mouth-breathing children were lower than in the nasal-breathing group.
Noor et al., 2021 [20].	Cross-sectional.	From children (6 years) to emerging adults (20 years).	Relationship between breathing patterns and the presence of malocclusions.	A higher percentage of mouth breathing was found in subjects with Class II and Class III malocclusion. The correlation between malocclusions and breathing patterns was not significant.
Festa et al., 2021 [21].	Descriptive.	Children.	Upper airway obstruction, mouth breathing, and malocclusions.	Mild tonsillar hypertrophy was found to be significantly associated with Class II malocclusion and increased horizontal overbite in mouth-breathing children. These results may suggest an important role for malocclusion in the onset of childhood mouth breathing, or they may be an effect of tonsillar hypertrophy.
Sakai et al., 2021 [22].	Systematic review.	Children and adolescents who breathe through their mouths.	Rapid maxillary expansion to correct transverse maxillary deficiency in mouth breathers.	Children and adolescents treated with Rapid Maxillary Expansion showed an increase in nasomaxillary structures such as the nasal cavity, oropharynx, nasopharynx, maxillary sinuses, maxillary width, and dental arches. These results led to an improvement in mouth breathing.
Lysy et al., 2021 [23].	Retrospective.	Children from 9 years to adults of 47 years.	Dentofacial characteristics: maxilla (Mx), intercanine distance (MxIC), buccal cusp of first premolars (MxIP), distance between the mesial groove of first molars (MxIM), mandible (Mn), cephalometric analysis (ANB, SNA, SNB, PP-MP, SN-MP, N-S-Ba, ArGoMe) and mouth breathing.	The angles ANB, SNA, SNB, SN-Ba, PP-MP, and SN-MP, and the widths MxIC, MnIC, MnIP, and MnIM, did not differ in mouth breathers. The ArGoMe angle was found to be increased in mouth breathers, and the MxIM width showed significant differences.
Zárate et al., 2025 [8].	Systematic review.	Subjects with transverse maxillary deficiency aged 9 to 37 years.	Structural changes in the craniofacial complex induced by a skeletal expander supported by a microimplant.	Studies demonstrate significant structural changes in the craniofacial complex following microimplant-supported skeletal expansion (MSE). The technique promotes orthopedic expansion with fewer adverse dental and periodontal effects compared with non-skeletal expansion approaches.
Satiti et al., 2025 [24].	Cross-sectional.	Children aged 10 to 12 years.	Maxillary transverse dimensions MWM, IMW, MWC and ICW, airway volume and mouth breathing.	Mouth-breathing children presented significantly reduced maxillary transverse dimensions; the MWM, IMW, MWC, and ICW parameters were notably narrower, indicating a constriction of the dental arch. Furthermore, the nasopharyngeal and oropharyngeal volumes were smaller, and the cross-sectional area of the nasopharyngeal and oropharyngeal regions was reduced.
Kumari et al., 2025 [25].	Cross-sectional.	Children aged 6 to 14 years.	Cephalometric evaluation using lateral cephalometric radiography and mouth breathing.	In children who breathe through their mouths, there is a high prevalence of retrognathic jaws (71.6%), vertical growth tendencies (29.5%), and skeletal discrepancies. A Class I skeletal pattern was identified in 54.5% and a Class II pattern in 42.0%.

Note: Because the literature reviewed featured varied study designs and reporting approaches, details such as sample size, diagnostic criteria for mouth breathing, and methods for craniofacial assessment were not consistently documented.

## Data Availability

No new data were created or analyzed in this study. Data sharing is not applicable to this article.
